# Safety of ilunocitinib tablets (Zenrelia™) after once daily oral administration in dogs

**DOI:** 10.1186/s12917-025-04579-1

**Published:** 2025-03-05

**Authors:** Emmanuelle A. Kuntz, Les Gabor, Céline E. Toutain

**Affiliations:** 1 Elanco Animal Health, Mattenstrasse 24a, Basel, 4058 Switzerland; 2 Elanco Animal Health, Yarrandoo R&D Centre, Kemps Creek, Sydney, 2178 Australia; 3 Elanco Animal Health, Crisco Uno, Bâtiment C, 3-5 avenue de la Cristallerie, CS 80022-92317, Sèvres, Cedex France

**Keywords:** Ilunocitinib, Zenrelia™, Safety, Dog, Oral

## Abstract

**Background:**

Ilunocitinib is a new molecular entity of the Janus kinase inhibitor (JAKi) class for the treatment and control of symptoms of allergic skin disease conditions, such as pruritus and skin lesions in dogs. This laboratory study with ilunocitinib tablets (Zenrelia™, Elanco) investigated the safety in healthy dogs treated once daily for 6 months. The study was a randomized, blinded, parallel-group design examining one (1X), two (2X), three (3X) and five (5X) times the maximum recommended dose (0.8 mg/kg) compared to sham dosed control dogs.

**Methods:**

Twenty male and 20 female healthy Beagle dogs, 11 to 12-month of age, with a mean body weight ranging from 9.85 to 10.46 kg, were randomized to an untreated control group or ilunocitinib treatment groups at daily dose rates of 0.8 mg/kg (1X), 1.6 mg/kg (2X), 2.4 mg/kg (3X), or 4.0 mg/kg (5X) over six months. All animals were fed within 30 min prior to treatment. Safety assessments included general health observations, clinical observations (including complete physical and neurological examinations), ophthalmology, clinical pathology, peripheral blood immunophenotyping, body weight, food consumption, pharmacokinetic blood collections, organ macroscopic and microscopic examinations.

**Results:**

No effects of the treatment were noted on body weight, food consumption, physical and neurological examinations, urinalysis, peripheral blood immunophenotype, and ophthalmoscopic examinations. Clinical findings included non-exudative skin lesions, skin lesions with discharge, swollen paw(s), skin thickening, skin discoloration or scabbing of the feet (paws/digits), in both male and female dogs. Changes in clinical pathology included marginally decreased red cell mass, lower eosinophils, higher C-reactive protein, total protein and fibrinogen, lower albumin and albumin: globulin levels. Microscopic findings included skin inflammation and focal dermatitis/furunculosis.

**Conclusions:**

Ilunocitinib was well tolerated when administered daily over six months at 0.8 mg/kg and multiples thereof up to 4.0 mg/kg in Beagle dogs. At the therapeutic dose, no clinically significant changes were observed. Minimal changes in hematological parameters, total protein, and fibrinogen were noted at the higher doses. All of these findings, consistent with the known pharmacology of the JAKi class at exaggerated dosing, support the safe and chronic use of Zenrelia™ tablets.

**Clinical trial number:**

Not applicable.

## Background

Skin disorders rank as one of the most frequent reasons for pet owners to seek veterinary care, with pruritus being a particularly distressing symptom significantly diminishing the quality of life for both dogs and their owners [1]. Atopic dermatitis is a prevalent inflammatory condition affecting roughly 15% of the canine population. It is a primary cause of pruritus, and one of the most frequent underlying causes of presentation to veterinary practitioners [2, 3]. The pathogenesis of canine atopic dermatitis is complex. It involves genetic factors, a dysfunction of the skin barrier, a disruption of the skin microbiota, immune dysregulation, and allergic sensitization [4]. In dogs, studies have shown a strong correlation between atopic dermatitis and genetic mutations. However, the disease is multifactorial, and the environment plays an important role. Other notable causes of pruritus include flea allergy dermatitis, food allergies, and sarcoptic mange. Pruritus is not merely a symptom; it exacerbates allergic conditions by repeatedly disrupting the skin barrier, thus facilitating the entry of allergens, bacteria, and yeasts. Therefore, addressing pruritus is not just symptomatic relief but also serves as an etiological treatment strategy.

The pharmacological landscape for managing allergic and pruritic conditions in dogs includes several drug families such as glucocorticoids, calcineurin inhibitors (e.g., Cyclosporin A), Janus Kinase inhibitors (JAKi), and anti-IL-31 monoclonal antibodies (mAb). While many of these treatments are effective, they may come with undesirable side-effects, such as those observed with glucocorticoids, or may alleviate pruritus without addressing underlying inflammation, as with anti-IL-31 mAb. The veterinary market has been limited to a single JAKi, oclacitinib, for the treatment of canine atopic dermatitis and associated pruritus, while human medicine has access to a broader range of JAKi for a wide range of immune mediated conditions [5, 6].

Ilunocitinib, a novel JAKi, has recently been introduced to the veterinary field as Zenrelia™, offering a new option for managing pruritus associated with allergic dermatitis and atopic dermatitis in dogs. Studies have demonstrated its efficacy with pharmacological properties suitable for once-daily dosing, ensuring sustained therapeutic levels [7–9].

This study aimed to assess the safety profile of ilunocitinib in dogs when given once daily, over 6 months at 0.8 mg/kg (1X) and multiple overdose levels (1.6 (2X), 2.4 (3X) and 4.0 (5X) mg/kg). Furthermore, the safety of any new veterinary medication must be evaluated in the target species to meet regulatory requirements.

## Methods

### Regulatory and ethical compliance

This laboratory study was conducted with reference to the guideline for evaluating the target animal safety of new pharmaceuticals (VICH Guideline 43) [10], and in accordance with Good Laboratory Practice [11–12]. The study protocol was reviewed and approved by the Testing Facility Institutional Animal Care and Use Committee and was conducted in compliance with the Final Rules of the Animal Welfare Act regulations and the Guide for the Care and Use of Laboratory Animals [13–14]. This manuscript was prepared in compliance with the ARRIVE Guidelines Checklist for animal in vivo experiments [15].

### Animal management

A total of 22 male and 22 female experimentally naïve Beagle dogs purchased from a laboratory animal supplier (Envigo USA) of approximately 11 to 12-months of age were acclimatized for approximately 3 weeks. Following veterinary examinations and clinical pathology evaluations, 20 males and 20 females (weighing 8.8 to 12.4 kg and 5.5 to 11.2 kg, respectively, at randomization) were selected and assigned to the control or treatment groups on study day − 1.

Dogs were pair-housed (2 animals/cage of same sex) from arrival until study day − 3 and were then individually housed for the duration of the study in stainless steel mobile cages with poly-coated diamond shaped flooring containing animal enrichment. Through the whole study duration, tap water was available *ad libitum* and animals were offered daily dry laboratory diet (Lab Diet^®^ Certified Canine Diet #5007) and a palatable wet canned food (Royal Canin Veterinary Diet Recovery RS). On dosing days (study day 1 to 182), dogs were treated in fed conditions i.e. dogs were fasted overnight and offered canned food 20–30 min before tablet administration. The daily ration of dry food was offered 1 to 2 h following dosing.

### Randomization, blinding and treatment

This was a randomized, controlled, blinded, 6-month study with a parallel design. Using a block randomization procedure stratified by sex, 20 males and 20 females were randomly allocated on study day − 3 to one of the following five groups (4 males and 4 females per group): Group 1: Untreated control (sham-dosed with 5 mL of tap water); Group 2: Ilunocitinib film-coated tablets at a target dose level of 0.8 mg/kg (1X); Group 3: Ilunocitinib film-coated tablets at a target dose level of 1.6 mg/kg (2X); Group 4: Ilunocitinib film-coated tablets at a target dose level of 2.4 mg/kg (3X) and Group 5: Ilunocitinib film-coated tablets at a target dose level of 4.0 mg/kg (5X). The experimental unit was the individual dog.

With the exception of individuals either administering the treatments or with roles unrelated to data collection, all other personnel were blinded to the treatment group assignments.

### Test article administration

The dose levels were selected based on the maximum therapeutic dose (i.e. 0.8 mg/kg, 1X) and the requirement to test multiples of the maximum therapeutic dose, i.e. 2X, 3X and 5X the therapeutic dose. Doses for each individual animal were based on the most recent body weight. Commercial tablets of ilunocitinib, available in 4.8, 6.4, 8.5, and 15 mg strength, were dispensed. These tablets were combined appropriately to meet the individual’s precise dosage requirement, ensuring there was no underdosing. The administration of these tablets was carried out orally, once a day, over a period of 6 months, from study day 1 to 182. Approximately 5 mL of water was given after each administration and the mouth checked to ensure the tablets had been swallowed. Control animals were sham treated with 5 mL of tap water. As ilunocitinib exposure has been shown to be increased by food, dogs were fed within 30 min prior to each dosing.

### Safety variables

Animals were observed twice daily, at least 6 h apart, over the entire course of the study for morbidity, mortality and injury. Clinical examinations of each dog were performed before treatment administration and at approximately 2 and 5 h post treatment. Observations included, but were not limited to, evaluation of skin, fur, eyes, ears, nose, oral cavity, thorax, abdomen, external genitalia, limbs and feet, respiratory and circulatory signs, autonomic effects such as salivation, and nervous system effects including tremors, convulsions, reactivity to handling, and unusual behavior.

Body weights for all animals were recorded during the acclimation phase (study days − 12 and − 1) and then once weekly during the study. Food consumption (dry and wet food) was measured and recorded daily. Complete physical examinations by a veterinarian were conducted once during acclimation and every two weeks during the dosing phase on study days − 6, 14, 28, 42, 56, 70, 84, 98, 112, 126, 140 154, 168 and 182. Assessments including overall general condition and behavior, ocular inspection, integument, musculoskeletal, gastrointestinal, body temperature, cardiovascular and respiratory systems including auscultation-based assessment, reproductive, lymphatic, urinary and nervous systems. Neurological examinations were conducted once during acclimation on study day − 6 and on study day 182 including evaluation of the general attitude, behavior, motor function, cranial nerves (nystagmus, pupillary response), proprioceptive and postural reactions, and spinal nerves. Ophthalmoscopic examinations were conducted by a board-certified ophthalmologist on all animals on study days − 4 and 182.

Blood samples were collected from the jugular vein for the evaluation of hematology, clinical chemistry and coagulation variables at pre-test, and on study days 29, 57, 85, 113, 141, 169 and 183. The complete panel of clinical pathology parameters (hematology, blood chemistry and urinalysis) according to VICH Guideline 43 [10] are listed in Table 1. Urine samples were collected using steel pans placed under the cages for at least 16 h at the same timepoints.

**Table 1:** Clinical pathology parameters

Hematology	Coagulation	Clinical chemistry	Special chemistry	Urinalysis
Red blood cell count	Prothrombin time	Alkaline phosphatase	C-reactive protein	Volume
Hemoglobin concentration	Activated partial thromboplastin time	Total bilirubin		Color
Hematocrit	Fibrinogen	Aspartate aminotransferase		Appearance
Mean corpuscular volume		Alanine aminotransferase		Specific gravity
Red blood cell distribution width		Gamma glutamyl transferase		pH
Mean corpuscular hemoglobin concentration		Urea nitrogen		Protein glucose
Mean corpuscular hemoglobin		Creatine kinase		Bilirubin
Reticulocyte count (absolute and percent)		Creatinine		Ketones
Platelet count		Total protein		Blood
Red blood cell distribution width		Amylase		Urobilinogen
White blood cell count		Lactate dehydrogenase		
Neutrophil count (absolute and percent)		Bile acids		
Lymphocyte count (absolute and percent)		Albumin		
Monocyte count (absolute and percent)		Globulin (calculated)		
Eosinophil count (absolute and percent)		Albumin/globulin ratio (calculated)		
Basophil count (absolute and percent)		Glucose		
Large unstained cells (absolute and percent)		Total cholesterol		
Other cells (as appropriate)		Triglycerides		
		Electrolytes (sodium, potassium, chloride)		
		Calcium		
		Phosphorus		
		Magnesium		

Whole blood samples were also collected from the jugular vein for the evaluation of the leukocyte subsets and phenotypes on study days − 16 and − 8, and on study days 29, 57, 85, 113, 141, 169 and 183. The immunophenotype profiling by flow cytometry included total T lymphocytes (CD45 + CD5 + or CD5 + T cells), Helper T lymphocytes (CD45 + CD5 + CD4 + or CD4 + Th cells), Cytotoxic T lymphocytes (CD45 + CD5 + CD8 + or CD8 + Tc cells), B lymphocytes (CD45 + CD5- CD21 + or CD21 + B cells) and monocytes (CD45 + CD14+).

### Blood samples for pharmacokinetic analysis

Blood samples (1 mL) were collected from all animals via the jugular vein for determination of the plasma concentrations of ilunocitinib. Samples were collected at pre-dose and at 0.5, 1, 2, 5, 8 and 24 h post-dosing on the day of the first and last dose (study days 1 and 182) and at pre-dose and at 1 and 24 h post-dosing at study day 85. Blood samples were collected in tubes containing K_2_ EDTA. Blood was processed to plasma within one hour of collection and plasma samples were stored frozen at -20 °C until analyzed. Plasma concentrations of ilunocitinib were determined using a validated Liquid Chromatography-Tandem Mass Spectrometry (LC-MS/MS) method according to Guidance for Industry [16–17].

The bioanalytical data were processed as individual concentration vs. time profiles using a non-compartmental analysis (NCA). Calculations were performed with the software Phoenix WinNonlin (version 8.3; Certara, USA). Tmax and Cmax were derived from observed values. AUCs were calculated using the Linear Up Log Down method. In addition, T1/2 was calculated using the best fit method and dose, time and sex effects were evaluated based on Cmax and AUClast ratios.

### Gross and microscopic evaluations

At study termination (study day 183), the dogs were humanely euthanized by an intravenous injection of pentobarbital sodium/phenytoin solution under sedation with 6.6–13.2 mg/kg Telazol^®^ (tiletamine/zolazepam) subcutaneous injection. Gross and microscopic examinations were carried out on all animals by a board-certified veterinary pathologist.

### Statistical methods

All statistical analyses were performed using SAS^Ⓡ^ software (Version 9.4, TS1M6 SAS/STAT 15.1, Copyright© 2002–2012 by SAS Institute Inc., Cary, NC, USA).

Endpoints measured once post-treatment (organ weights) that did not include a pre-treatment measurement were analyzed using analysis of variance (ANOVA) with ‘treatment’, ‘sex’, and ‘treatment by sex’ as fixed effects.

Endpoints measured multiple times post-treatment that included a pre-treatment measurement (Serum chemistry, C-reactive protein, coagulation, hematology, urinalysis, body weight, body weight changes and food consumption) were analyzed using repeated measures analysis of covariance (RMANCOVA) with ‘treatment’, ‘time’, and ‘sex’; the two-way interactions ‘treatment by time’, ‘treatment by sex’, and ‘sex by time’; the three-way interaction ‘treatment by time by sex’ and a covariate all as fixed effects. The last available pre-treatment value was used as the covariate.

If the interaction terms were significant (*P* ≤ 0.10 level for two-way interactions and *P* ≤ 0.05 for the three-way interaction), treated groups were compared to the control either within each sex (treatment by sex significant), within each time point (treatment by time significant) or main effect only (neither treatment by sex nor treatment by time significant).

For immunophenotyping/peripheral blood leukocytes, a generalized linear mixed model for repeated measures analysis of variance (mixed model) was conducted. For each endpoint, the model tested for the effects of treatment, time, sex and all two and three-way interactions. The last available pre-treatment value was included in the model as a covariate. The evaluation proceeded as described for RMANCOVA.

## Results

### Dose administration

During the dosing period of the study, the mean doses of ilunocitinib administered for 1X, 2X, 3X, and 5X, were 0.83, 1.63, 2.43 and 4.03 mg/kg, respectively and the target dose rate planned was achieved for all treated animals on all days.

### Clinical observations

All animals were in good health throughout the duration of the study and completed the study. There were no serious adverse effects during the course of the study.

Test article-related clinical observations revealed a dose-dependent presence of interdigital furunculosis, or cysts (Table 2), with or without discharge on one or more paws. Lesser cutaneous lesions such as occasional swelling or scabbing of paws, along with thickening and/or discoloration of paw skin were also noted.

No ophthalmological or neurological findings were associated with treatment.

**Table 2:** Summary of clinical and histological findings on number of animals affected in the study groups

	0X	1X	2X	3X	5X
Skin discoloration	0	1	1	2	0
Skin lesion	2	4	5	6	8
Skin lesion w/discharge	1	1	3	3	4
Swollen paws	1	1	5	6	3
Skin thickening (paws)	1	0	1	1	0
Skin scab paw	2	0	0	1	1
Papilloma	0	0	0	1	2
Folliculitis/furunculosis	0	0	0	0	3

### Body weights and food consumption

Throughout the study, the mean body weight (± standard error) of control dogs was 9.85 ± 0.20 kg, while the mean body weights in the treatment groups were as follows: 10.30 ± 0.20 kg in the 1X group, 9.76 ± 0.20 kg in the 2X group, 10.31 ± 0.20 kg in the 3X group, and 10.46 ± 0.20 kg in the 5X group. Body weight gain for the control dogs was 0.03 ± 0.02 kg, compared to 0.06 ± 0.02 kg in the 1X group, 0.03 ± 0.02 kg in the 2X group, 0.06 ± 0.02 kg in the 3X group, and 0.06 ± 0.02 kg in the 5X group. A statistically significant main effect of treatment was observed for body weight (*p* *= 0.0026*) with statistically significant increases in the 1X, 3X and 5X groups. A statistically significant main effect of treatment was observed for body weight change (*p* *= 0.0952*) with statistically significant increases at 3X and 5X. The weekly averaged daily food consumption showed a statistically significant (*p* *= 0.0872*) time by treatment interaction.

### Clinical pathology

**Fig. 1:**
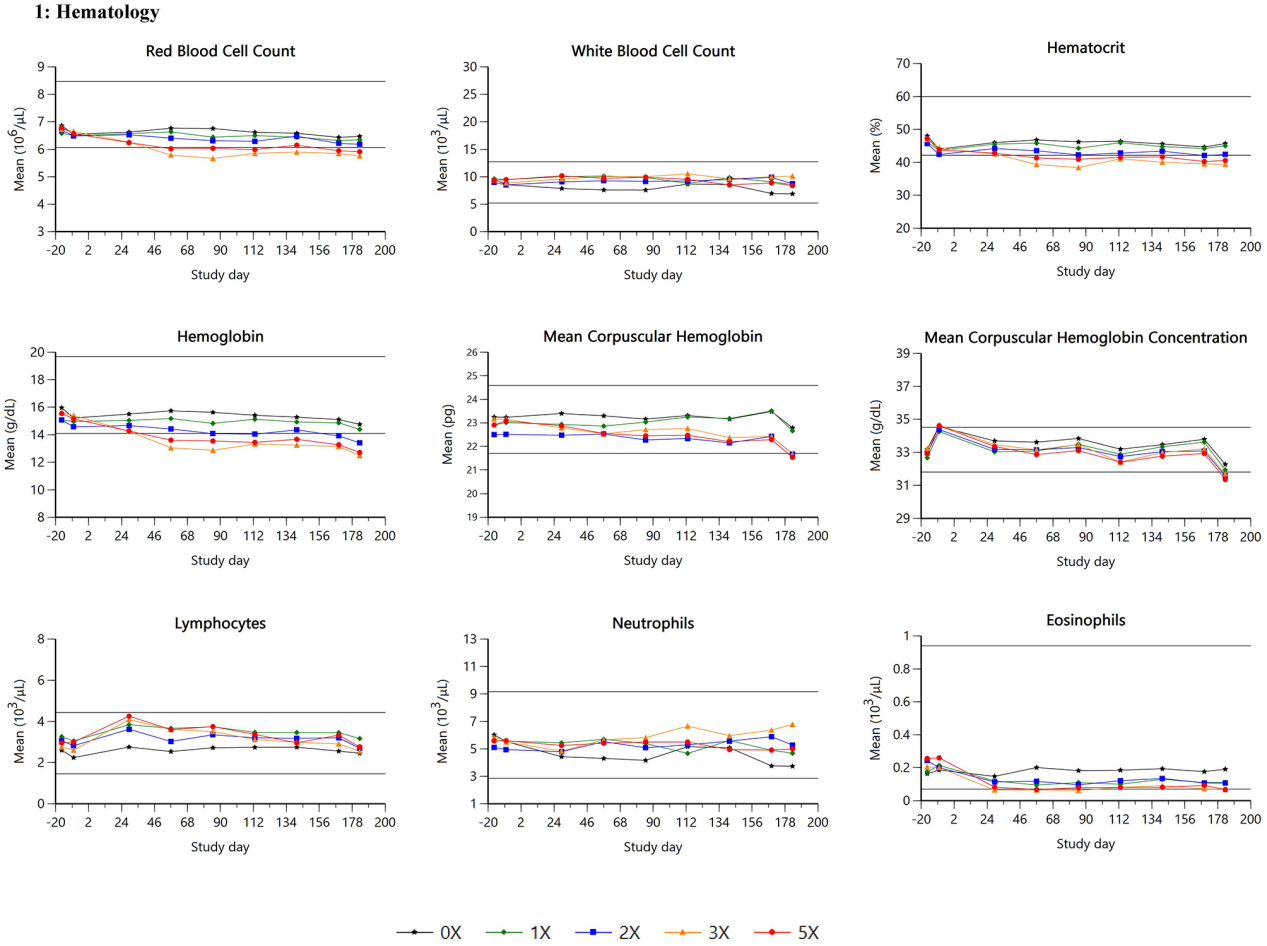
Hematology

**Fig. 2:**
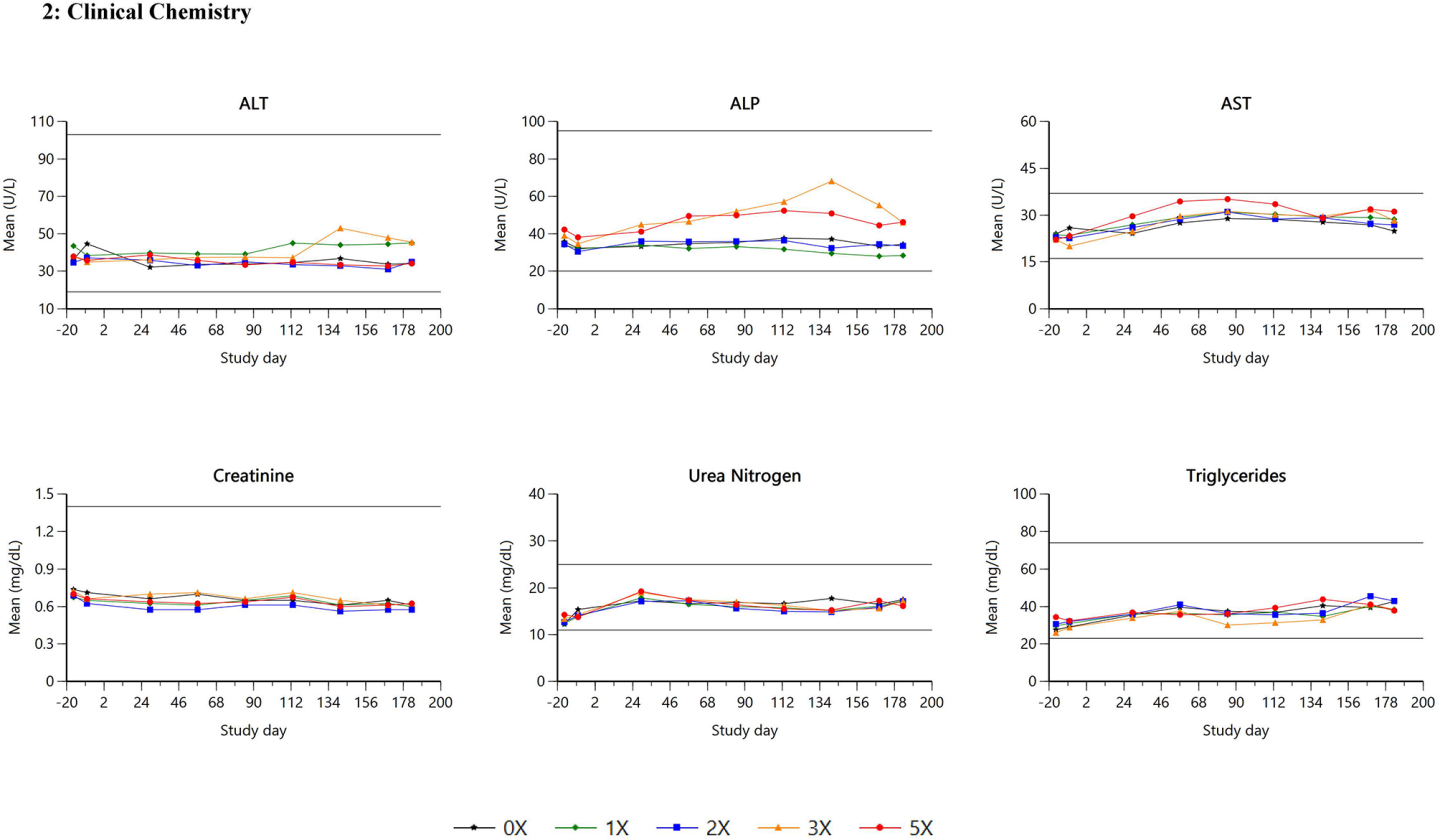
Clinical chemistry

**Fig. 3:**
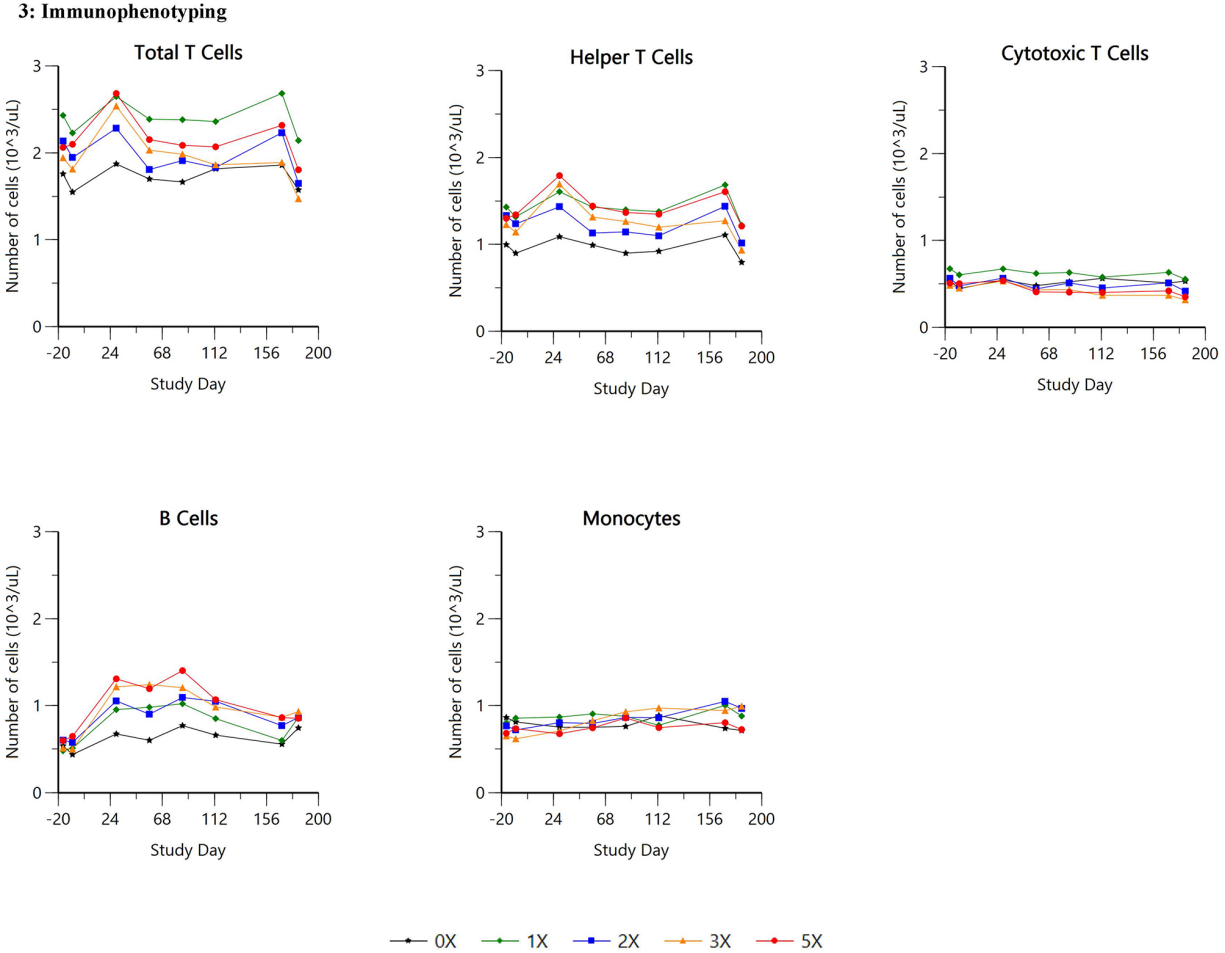
Immunophenotyping

Summary data for selected hematology and clinical chemistry parameters are presented in Table 3; Figs. 1, 2 and 3.

**Table 3:** Summary of Ilunocitinib main effects on clinical pathology parameters

Test Article Effects Compared to 0X						
	**Study day**	**Groups significantly impacted**				**Comments**
		**1X**	**2X**	**3X**	**5X**	
**Hematological parameter**						
HCT	all			↓	↓	
Hb	all		↓	↓	↓	
RBC	all			↓	↓	
MCH	all			↓	↓	
MCHC	all		↓	↓	↓	
Eosinophils	29–183			↓	↓	*females only*
**Clinical chemistry parameter**						
Total Protein	all		↑	↑	↑	*significant in pooled by sex; males (2X*,* 3X*,* 5X) females (3X*,* 5X)*
	113			↑		
	85,113,141				↑	
Albumin				↓	↓	*significant in pooled by sex; males (2X*,* 3X*,* 5X) females (3X*,* 5X)*
**Coagulation parameter**						
Fibrinogen	57–183			↑	↑	*association with interdigital cysts consistent with acute phase response*

Statistically significant treatment effects for hemoglobin (*p* < 0.0001), hematocrit (*p* < 0.0001) and red blood cell count (RBC; *p* = 0.0001) were noted reflecting minimally lowered mean hemoglobin for pooled sexes at 2X, 3X, and 5X, minimally to mildly lower mean hematocrit and RBC for pooled sexes at 3X and 5X, relative to control means. These differences began from study day 29 and persisted through study day 183. Concurrently, there was a significant *time by treatment* interaction for mean corpuscular hemoglobin (MCH; *p* = 0.0260) and a significant treatment main effect for mean corpuscular hemoglobin concentration (MCHC; *p* < 0.0001) due to minimally lower mean MCH at 3X and 5X and minimally lower mean MCHC 2X, 3X and 5X.

Eosinophils showed a significant *sex by treatment* interaction (*p* = 0.0050) in females. For females, treatment groups at 3X and 5X had lower means compared to controls from study days 29 through 183. There were no treatment-related effects on lymphocytes, monocytes, basophils and reticulocytes. Two dogs in the 5X group had minimally lower myeloid: erythroid ratios.

There was a significant treatment effect for fibrinogen (*p* < 0.0001) at 3X and 5X doses, with marginally elevated concentrations from study days 57 to 183. The other coagulation parameters (prothrombin time and activated partial thromboplastin time) remained in the reference range throughout the study.

Total protein values were significantly (*p* = 0.0437) higher in males at 2X, 3X, and 5X and displayed a significant *time by treatment* interaction (*p* = 0.0245), characterized by minimally higher means on study day 113 at 3X and on study days 85, 113, and 141 at 5X.

There was a significant main effect of treatment for albumin (*p* = 0.0017), with minimally lower mean albumin concentration at 3X and 5X doses beginning on study day 57. Globulin and albumin/globulin ratio had significant 3-way interactions (*p* = 0.0005 and *p* < 0.0001, respectively). Mean globulin concentrations were minimally higher and mean albumin/globulin ratios were minimally to mildly lower than control means in males at 2X, 3X, and 5X and in females at 5X beginning on study day 57, 85, or 113 and persisting through study day 183. No changes were noted for alkaline phosphatase, alanine aminotransferase, aspartate aminotransferase, triglycerides, plasma creatinine and urea levels or any other clinical chemistry parameters listed in Table 1, all remaining within normal ranges for the treatment groups.

There were no test article-related effects on urinalysis endpoints in either sex at any dose level.

There were no test article-related effects on leukocyte analysis (immunophenotyping) parameters in either sex at any dose level.

### Organ weights, gross and microscopic examinations

There was a single main treatment effect related to reduced combined adrenal gland weights in males treated at 2X and 5X whereas males at 1X and 3X doses were not changed when compared to controls. In contrast, females at 3X had increased combined adrenal gland weight. Prostate weight (*p* = 0.0123), prostate weight as a percent of body weight (*p* = 0.0050) and the ratio of prostate weight to brain weight (*p* = 0.0279) were significantly decreased in the 5X group compared to controls.

Macroscopic observations affecting 11 animals across all groups were recorded at the end of the study. Two animals (0X and 2X) exhibited unilateral thickening of the left ear pinnae, consistent with focal trauma and were considered not treatment-related. There were low numbers of incidental findings, including a single case of enlarged thyroid/parathyroid glands in one female treated at 1X, and two focal lesions on the lungs of animals treated at 5X.

The remaining six animals showed only skin changes, some with enlarged draining lymph nodes, interpreted as normal responses to cutaneous inflammation. One animal (3X) had four raised dermal nodules on each paw, consistent with papillomas and associated with *Demodex* spp. fragments and follicular cysts. Five animals in the highest dose group (5X) had various skin lesions, primarily papillomas or dermatitis/furunculosis, with reactive lymph nodes.

Microscopic findings across all groups were minimal and largely consistent with background pathology, with no adverse effects noted except for cutaneous changes. In the highest dose group (5X), cutaneous lesions were observed in 5 out of 8 animals. These included various feet skin lesions such as papillomas in 2 animals and inflammatory lesions with hair fragments consistent with folliculitis/furunculosis in 3 animals (Table 2). Some animals also displayed reactive lymph nodes. The cutaneous lesions in the 5X group were interpreted as treatment-associated.

### Pharmacokinetic results

Plasma concentrations were quantifiable at all timepoints in all dogs across the four dosing levels (0.8, 1.6, 2.4, or 4.0 mg/kg) on study days 1, 85, and 182. Pre-dose concentrations on study days 85 and 182 and concentrations 24 h after dosing on study days 1, 85, and 182 were consistently low, indicating low accumulation after daily administrations.

Pharmacokinetic parameters were calculated for all treated dogs on study day 1 and study day 182 and are summarized in Table 4 with respect to dose level and treatment day. The variability of the pharmacokinetic parameters Cmax and AUCs was generally low or moderate. The half-life was approximately 3 h across all groups on days 1 and 182.

**Table 4:** Summary of pharmacokinetic parameters administered orally once daily for 6 months at 4 dose levels

	Gp 2 (1X: 0.8 mg/kg)				Gp 3 (2X: 1.6 mg/kg)				Gp 4 (3X: 2.4 mg/kg)				Gp 5 (5X: 4.0 mg/kg)			
	Day 1		Day 182		Day 1		Day 182		Day 1		Day 182		Day 1		Day 182	
Tmax (h)*	2	(1–2)	2	(1–2)	1.50	(1–2)	2	(1–2)	2	(1–2)	2	(1–5)	2	(2–2)	2	(1–2)
Cmax (ng/mL)	274	(24.2)	310	(20.6)	433	(21.0)	540	(13.4)	659	(15.1)	972	(33.2)	919	(15.6)	1080	(29.4)
Cmax/D (ng/mL)**	327	(23.0)	378	(20.6)	269	(21.7)	332	(13.6)	272	(15.5)	400	(33.7)	227	(16.3)	270	(29.1)
AUClast (h*ng/mL)	1150	(25.3)	1360	(25.1)	1710	(24.3)	2440	(21.3)	3310	(28.5)	5190	(52.4)	4380	(14.7)	5420	(22.6)
AUClast/D (h*ng/mL)**	1370	(26.4)	1660	(25.8)	1060	(24.9)	1500	(20.5)	1360	(29.0)	2130	(52.9)	1080	(14.8)	1350	(22.3)
AUCinf (h*ng/mL)	1160	(25.8)	1370	(25.5)	1720	(24.5)	2450	(21.0)	3360	(29.1)	5140	(59.0)	4410	(14.8)	5470	(23.1)
AUCinf/D (h*ng/mL)**	1380	(26.9)	1670	(26.2)	1070	(25.0)	1510	(20.2)	1380	(29.6)	2110	(59.6)	1090	(14.8)	1360	(22.8)
AUCextra (%)	0.595	(77.4)	0.557	(70.9)	0.398	(54.3)	0.472	(118)	0.918	(173)	0.891	(215)	0.498	(54.6)	0.557	(133)
T1/2 (h)	3.36	(14.6)	3.29	(11.9)	3.08	(8.95)	2.68	(28.5)	3.68	(24.8)	3.62	(27.4)	3.08	(10.6)	3.20	(20.7)

Geometric means and (geometric CV%) except for (*) Tmax where the median and range (minimum-maximum) are given

** Cmax and AUC are dose normalized to 1 mg/kg

Exposure increased with dose and the increase was close to dose proportional (Table 4). Exposure was slightly higher at study day 182 with AUC ratio of 1.2, 1.4, 1.6, and 1.2 for groups 2 to 5 respectively (Table 4). Exposure was similar in males and females for each treatment group and day, demonstrating absence of any significant differences between males and females.

## Discussion

The objective of this study was to investigate the safety of chronic daily oral administration of a novel JAKi, ilunocitinib, in dogs at the therapeutic dose rate (0.8 mg/kg) and overdoses (1.6, 2.4 and 4.0 mg/kg). The conduct of this study is a requirement in order to register a new veterinary medicinal product. It is an integral part of the safety data package, providing substantial evidence of a product’s safety. The design of the study including selection of dose levels, number of animals and endpoints were chosen in accordance with VICH Guideline 43, with the objective to establish a margin of safety [10]. To date, only one other JAKi is licensed for dogs (oclacitinib). To the authors’ knowledge, there is no other publication of safety data generated with any JAKi for veterinary use and with overdoses and only clinical safety data at the therapeutic doses are published.

The results demonstrated that ilunocitinib was well-tolerated by dogs at the therapeutic dose (0.8 mg/kg), and clinically well tolerated at higher dose rates. During the course of the study, no serious adverse effects or mortality occurred, including at 5X overdose for 6 months, and all treated animals completed the planned study duration.

Although statistical significance in body weight and body weight changes was observed compared to the controls, no dose-dependent relationship was detected. Moreover, the results consistently remained slightly above control values, within established reference ranges, and lacked clinical relevance. The impact on body weight may vary among JAK inhibitors depending on their selectivity for JAK subtypes. Weight gain has been reported in dogs with the use of oclacitinib [18] and in very few cases under field conditions with ilunocitinib [19]. In humans, the mechanism behind tofacitinib-induced weight gain has been reported to be linked to a reduction in JAK3 activity, which is implicated in obesity pathogenesis [20]. Baricitinib, a close chemical analog of ilunocitinib, exhibits significantly lower potency against JAK3, resulting in limited weight gain, thereby supporting its potential beneficial role in obesity and metabolic disease development [21].

Clinical findings were mainly related to skin lesions in the form of interdigital cysts (IDC) or interdigital furunculosis. Whilst cutaneous lesions were noted with increasing doses, they were mild and not unexpected with immune modulation. The finding of cutaneous papillomas, IDC or furunculosis is known in research or laboratory Beagle dogs, in which a higher than normal background incidence of IDC is present and generally exacerbated by the type of flooring used in the testing facilities together with an excess of moisture from daily cleaning procedures [22–23]. In a preliminary earlier research study, IDC were not observed during the course of the study when dogs were maintained on concrete floors with corn cob bedding (internal report). Immunosuppressants such as JAKis are known to increase the incidence of IDC in laboratory studies, but this effect has either not been observed or it has been seen only at a low incidence in clinical trials with client-owned dogs [24]. These findings are consistent with the known JAKi class effect of increased susceptibility to localized infections. While the incidence of the lesions increased with dose, they were mild and resolved by standard treatments.

Consistent with the clinical findings, microscopic analysis of the skin revealed the presence of papillomas in the high dose group. Papillomas are benign growths caused by the canine papillomavirus that generally result in little apparent discomfort or complications and are self-limiting. Papillomas were also observed with oclacitinib and resorbed spontaneously [25]. These conditions are common in the general canine population, particularly in laboratory dogs. The papillomas seen in this study are consistent with exophytic papillomas, which tend to be of viral origin, occur most commonly in dogs under 2 years of age, and regress spontaneously. In the current study, one of the cases of papilloma present on one paw in one single dog, was also associated with fragments of *Demodex canis*. *D. canis* mites are part of the normal flora of canine skin and usually cause no clinical disease. *Demodex* infection is a known potential effect of JAK inhibition secondary to immunosuppression [24–25]. Overall, the cutaneous findings were considered to have limited adversity, with most cases being sub-clinical and clinical cases typically responding well to routine care.

Treatment with ilunocitinib was associated with mild effects on hematology parameters associated with red cell mass. Similar variations in hematological parameters were typically reported for other JAKi used in human patients with rheumatoid arthritis [26] and in dogs treated with JAKi therapy [24]. The pattern of those alterations remains generally minor to moderate and is potentially only seen within a short period of time. Despite similarities in the pattern, differences in the effects were noted between JAKi used in human medicine. Treatment with baricitinib showed an initial decrease in hemoglobin within the normal range followed by a slight increase, while some other JAKi such as upadacitinib had limited impact on hemoglobin [27]. Ilunocitinib treatment induced mild decreases in red blood cell parameters (hemoglobin, hematocrit, RBC, MCH and MCHC), and eosinophils. The effects were dose-dependent and consistent with the known pharmacodynamic effects of JAK inhibition, potentially affecting erythropoiesis and eosinophil differentiation and activation. The decreases were minimal to mild in the overdose groups. In contrast, red cell parameters within the 1X group stayed within the reference range (Fig. 3). Slightly lower eosinophil counts are unlikely to have biological relevance. Regarding bone marrow, lower myeloid: erythroid ratios were reported to be treatment-related in two dogs and were consistent with a physiologically normal bone marrow response to the lower red blood cell mass despite no apparent effect on absolute reticulocyte counts. No main treatment effect on other cell lineages were observed, suggesting a relatively selective effect on specific JAK-mediated pathways.

There were minimal increases in fibrinogen at elevated doses, however, the clinical chemistry profile indicated individually elevated total protein and globulin levels, accompanied by reduced albumin levels and albumin/globulin ratios. C-reactive protein (CRP) levels were also higher. Notably, all dogs exhibiting these elevations also presented with visible skin disease. These findings were typical of an acute phase response and were temporally associated with microscopic inflammatory changes in the skin, presumed to be secondary to interdigital furunculosis. No further relevant changes were observed in clinical pathology parameters.

There was no main treatment effect of ilunocitinib on overall leukocyte cell populations (CD5 + total T lymphocytes, CD4 + Th cells, CD8 + Tc cells, CD21 + B cells (CD45 + CD5- CD21+) and monocytes). The lack of significant effects observed in this study is not surprising and mirrors a recent human study involving peripheral blood leukocytes phenotype analysis from healthy volunteers treated with tofacitinib. In this paper, changes were minor and reversible [28]. Similarly, baricitinib treatment in rheumatoid arthritis patients involved in phase III clinical trials showed similar minimal and reversible changes in lymphocytes subsets populations [29]. Most JAKi’s exhibit limited impact on lymphocyte populations due to their mechanism of action targeting intracellular signaling pathways rather than directly affecting cell surface receptors or differentiation pathways. The findings of this study provide evidence that ilunocitinib does not significantly alter immune cell populations at the therapeutic dose. This suggests a favorable safety profile regarding potential immune suppression, a concern often associated with immunomodulatory drugs.

Organ weight changes were minimal, not dose dependent or correlative with histopathological findings, thus, they were concluded to be of no biological relevance at the therapeutic dose. While recent studies showed that the JAK/STAT pathway is critical for metabolic organs such as adipose tissue, liver and pancreas [30], the study did not reveal any effect on main metabolic target organs, such as liver, kidney or primary lymphoid organs. Histopathology of all organs remained unremarkable with the exception of the cutaneous changes noted. Liver function, as assessed by enzymes including alkaline phosphatase, alanine aminotransferase, and aspartate aminotransferase, remained unaffected. Biochemical markers such as plasma creatinine and urea levels, along with urinalysis, were within normal ranges for the treatment groups. The potential concerns of a treatment with an immunosuppressant leading to promote or exacerbate different organ damage has not been observed when using ilunocitinib at the recommended therapeutic dose and overdoses.

The blood sampling schedule for pharmacokinetic assessment was designed to monitor drug exposure, while avoiding interference with the evaluation of primary safety endpoints. To maximize systemic exposure, the tablets were administered while the dogs were fed, since there is an approximate 20% increase in oral bioavailability of ilunocitinib when given with food or after feeding.

The inter-individual variability was generally low to moderate (CV < 30%) and consistent across groups for the PK parameters Cmax and AUCs. Residual concentrations 24 h post-dosing on study days 1, 85, and 182, as well as pre-dose concentrations on study days 85 and 182, were generally low, suggesting limited accumulation. This aligns with expectations considering the drug’s half-life, a fact further corroborated by the moderately increased exposure observed on study day 182.

Exposure exhibited a near dose-proportional increase, and no significant differences were detected between sexes, indicating a consistent and predictable response to the drug across different doses and sexes. Altogether, the pharmacokinetic analysis confirmed that the dogs were consistently dosed and exposed to ilunocitinib, supporting conclusions regarding the product’s safety.

While the study provides insights into the safety of ilunocitinib in dogs at overdosing to identify a margin of safety, it was conducted in a controlled laboratory environment using a relatively small number of healthy Beagle dogs. Consequently, the findings may not fully represent the variability seen in a broader canine population under field conditions. Factors such as pre-existing health conditions, different breeds, and environmental influences can impact the drug’s safety profile in real-world settings. Three clinical studies have been conducted, to further support the safety and efficacy of ilunocitinib in diverse canine populations in Europe and in the United States [6–8].

## Conclusions

In conclusion, this study provides comprehensive safety data on the use of ilunocitinib, a novel JAKi, in dogs. The study was carried out in accordance with the VICH Guideline 43 and aimed to establish a safety margin for the new veterinary medicinal product. Our findings indicate that ilunocitinib was well-tolerated in dogs at the therapeutic dose and even at doses up to five times higher, with no serious adverse effects or mortality observed.

The main clinical finding was a dose-dependent increase in interdigital cysts (IDC), an outcome consistent with the known JAKi class effect of increased susceptibility to localized infections. IDC could also be due to the type of flooring used in the testing facilities and were not observed when dogs were housed on cement and used corncob bedding. However, these lesions were generally mild and resolved by standard treatments. Additionally, papillomas, benign growths caused by the canine papillomavirus, were observed but are common in the general canine population and usually resolve spontaneously.

Ilunocitinib-related changes included mild decreases in red blood cell parameters and eosinophils in the high dose groups, consistent with the known pharmacodynamic effects of JAK inhibition, while the therapeutic dose group stayed within the normal range. No clinically relevant changes were seen in leucocytes cell populations, indicating limited effects on immune cell populations.

Overall, the results of this study suggest a favorable safety profile for ilunocitinib, supporting its chronic use in dogs.

## Data Availability

No datasets were generated or analysed during the current study.

## Abbreviations

ANOVA: Analysis of variance
ARRIVE: Animal research reporting in vitro experiments
AUC: Area under the curve
AUCextra (%): Portion of AUC that is extrapolated beyond the last measurable concentration to infinity
AUCinf: AUC from time zero to infinity
AUClast: AUC from time zero to the last measurable concentration
Cmax: Peak blood concentration
IDC: Interdigital cysts
JAK: Janus kinase
JAK/STAT: Janus kinase/signal transducer and activator of transcription
JAKi: Janus kinase inhibitor
MCH: Mean corpuscular hemoglobin
MCHC: Mean corpuscular hemoglobin concentration
RBC: Red blood cell count
RMANCOVA: Repeated measures analysis of covariance
T1/2: Half-life
Tmax: Time to reach peak blood concentration
VICH: Veterinary international conference on harmonization
